# White Spot Lesion Treatment Options: A Systematic Review of Different Techniques for Masking These Lesions

**DOI:** 10.3390/gels11050371

**Published:** 2025-05-19

**Authors:** Michela Lamorgese, Nélio Veiga, Maria J. Correia, Ana T. P. C. Gomes, Sara Lopes, Lígia Lopes-Rocha, Rita Fidalgo-Pereira, Pedro C. Lopes

**Affiliations:** 1 Faculty of Dental Medicine, Universidade Católica Portuguesa, 3504-505 Viseu, Portugal; lamo.michela@gmail.com (M.L.); nveiga@ucp.pt (N.V.); mcorreia@ucp.pt (M.J.C.); apgomes@ucp.pt (A.T.P.C.G.); snlopes@ucp.pt (S.L.); rfpereira@ucp.pt (R.F.-P.); 2 Center for Interdisciplinary Research in Health, Universidade Católica Portuguesa, 3504-505 Viseu, Portugal; 3 UNIPRO—Oral Pathology and Rehabilitation Research Unit, University Institute of Health Sciences (IUCS-CESPU), 4585-116 Gandra, Portugal; ligia.rocha@iucs.cespu.pt; 4 Department of Dental Sciences, University Institute of Health Sciences (IUCS), Cooperativa de Ensino Superior Politécnico e Universitário (CESPU), Rua Central de Gandra 1317, 4585-116 Gandra, Portugal

**Keywords:** white spot lesions, gels, fluoride, CPP-ACP, ozone therapy, remineralization, resin infiltration, minimally invasive dentistry

## Abstract

White spot lesions (WSLs) are early clinical stages of enamel demineralization, often related to orthodontic treatment or poor oral hygiene. The use of gels such as fluoride for topical application inhibits demineralization and promotes remineralization of dental tissues through various mechanisms. A variety of therapeutic approaches are available; however, recent research indicates that combined treatment strategies may yield superior clinical outcomes compared to monotherapy. The aim of this study was to critically compare the efficacy of combining multiple treatment techniques for WSLs compared to using these techniques alone. A systematic search was conducted in PubMed, Scopus, and Cochrane databases according to PRISMA guidelines. The PICO strategy was used to formulate the research question: Which clinical approaches combined or isolated (C) influence the treatment and prevention effectiveness (O) of white spot lesions (I) in humans (P) in the last ten years (T)? Inclusion criteria focused on clinical studies from the last ten years evaluating the combined use of at least two treatment techniques for WSL, resulting in a total of 8 randomized controlled trials selected from an initial pool of 1185 articles. Our results suggest that combined treatment strategies, including resin infiltration with fluoride varnish and ozone therapy combined with fluoride application, demonstrated enhanced efficacy in lesion masking and remineralization compared to single-treatment approaches. CPP-ACP and hydroxyapatite-based creams improved aesthetics, particularly when used alongside fluoride varnish. Our study concluded that the combination of remineralization agents like fluoride gel, infiltrative resins, and antimicrobial treatments offers superior outcomes on white spot lesion treatment than using these techniques alone. However, long-term clinical studies are needed to standardize treatment protocols and confirm durability.

## 1. Introduction

White spot lesions (WSLs) are an early manifestation of the caries process, also known as early caries lesions [1]. These lesions are characterized by changes in tooth enamel by mineral loss without cavities formation, signaling an imbalance between enamel demineralization and remineralization [2]. Although they do not necessarily proceed to caries [3], WSLs are an important risk indicator; these are the first visible manifestations of dental caries and can be reversed. WSLs are generally in the 1–2 range of the International Caries Detection and Assessment System (ICDAS II) [4]. Clinically, the appearance of the lesion is opaque white due to the optical phenomenon caused by mineral loss and the difference in the refractive index (RI) of water and air that fill the spaces formed in the enamel. The RI of healthy enamel is 1.62, matching with its primary component, hydroxyapatite. During enamel demineralization, the pore volume in WSLs increases, causing light to come across multiple interfaces with differing RIs, resulting in light scattering. The microporosities in these lesions are filled with either water (RI: 1.33) or air (RI: 1.0), and the difference in RI between sound enamel and these mediums leads to further refraction and scattering of light. The greater the disparity between the refractive indices, the brighter the lesion appears [5]. This optical phenomenon makes early carious lesions appear as clinically detectable white opacities, especially when the tooth surface is desiccated [6,7,8]. WSLs can have several etiological causes, including abnormalities in enamel development, fluorosis, trauma, orthodontic treatment, and inadequate oral hygiene [9].

The diagnosis of WSLs is mainly based on visual examination, which analyses the shape, size, color, and position of lesions, as well as depth assessment, a key factor in determining the most appropriate treatment [10,11]. The most common diagnostic method involves the combination of visual–tactile examination like the ICDAS, which is a diagnostic system [4] and bitewing radiography [12]. In addition to traditional methods, new diagnostic tools have been developed to improve lesion detection such as Electronic Caries Monitor (ECM) [13], Quantitative Light-induced Fluorescence (QLF) [14,15], and DIAGNOdent. However, diagnostic tools do not have the potential to change the form of treatment, whether it is a conventional method or a more differentiated one [11].

Several treatments are currently available for WSLs, from more invasive methods using bioactive gels, such as CPP-ACP (Casein phosphopeptide–amorphous calcium phosphate nanocomplex) and fluoride gel, to low molecular weight polymers conservative approaches such as and resin infiltrations [16]. Tooth-whitening gels can also improve the aesthetic appearance by reducing the contrast of WSLs [17,18,19]. This systematic review focused on these techniques, as it is essential for dentists to adhere to the principles of minimal intervention dentistry. By prioritizing non-invasive and/or micro-invasive strategies, practitioners can significantly reduce the loss of tooth structure [1,20,21].

Dental bleaching gels are one of the non-invasive methods applied in the treatment of WSLs. The efficacy of bleaching gels in the treatment of various types of discoloration depends on several factors, including severity, location, color, and depth of discoloration, as well as the number and duration of bleaching sessions, and the patient’s age [22]. However, a recent study showed that the use of 10% carbamide peroxide gel for bleaching can mask WSLs without affecting the chemical and mechanical properties of enamel [17]. Furthermore, the application of CPP-ACP has been suggested as a complementary treatment to promote remineralization of the sub-surface lesion [17]. In an in vitro study, Khoroshi et al. [23] demonstrated that a gentle and non-invasive bleaching procedure, which incorporates biomaterials such as nano-BAG, nano-hydroxyapatite, and nano-amorphous calcium phosphate within the bleaching agents can reduce the negative effects of bleaching and prevent enamel surface changes [23].

The resin infiltration technique involves the application of low-viscosity, light-curing resin on enamel caries lesions. The most popular commercial product (ICON^®^, DMG) contains, as its main component, the triethylene glycol dimethacrylate (TEGDMA) monomer, which, due to its high wettability, low viscosity, and low molecular weight of 286 g mol^−1^, penetrates WSLs by capillarity [24]. The WSLs’ treatment is achieved through the filling of WSLs; it strengthens and stabilizes demineralized enamel without compromising or sacrificing healthy tooth structure [8]. In addition, the properties of this infiltrative resin enable deep infiltration while preserving maximum tissue, making it a minimally invasive procedure that bridges the gap between preventive and conventional treatments. Once infiltrated and hardened, WSLs lose their whitish appearance, resembling healthy enamel, since it holds a refractive index of ~1.52, as apatite crystals [16]. Since the porosities of enamel caries act as diffusion pathways for dissolved acids and minerals, infiltration of these lesions with resin infiltrants could occlude these pathways, leading to the arrest of caries progression [25]. The infiltrative technique involves resin polymerization with a light-curing unit after an application period of three minutes [26]. Due to polymerization shrinkage, the infiltrating resin needs to be applied multiple times to ensure complete treatment of the lesion [21].

CPP-ACP is another alternative gel to treat initial enamel lesions or WSLs. It is a compound derived from casein [27], a milk protein capable of maintaining a high concentration of calcium, and phosphate ions on the tooth surface [28]. The remineralization model produced by CPP-ACP has been shown to significantly improve the aesthetics, strength, and acid resistance of remineralized WSLs. In fact, CPP-ACP can fill the enamel pores below the surface, decreasing the RI difference between demineralized porous enamel and healthy enamel, improving the translucency of the WSLs [27,29]. Another way, if not the most widespread, of controlling caries evolution and, consequently, WSLs from caries is by applying fluoride gel, especially with toothpaste [30]. Fluoride, which has been used in dentistry for over 70 years, is considered the main contributor to the decrease in caries prevalence globally [31]. It inhibits demineralization and promotes remineralization of dental tissues through various mechanisms. In hard tissues, fluoride forms a calcium fluoride layer on the tooth surface, which acts as a fluoride deposit and contributes to the formation of fluorapatite, a mineral that is poorly soluble in an acidic environment [31]. Although fluoride-enhanced remineralization is an effective non-invasive treatment for stopping WSLs, the aesthetic result may not be satisfactory. In general, WSLs remain visible due to the rapid precipitation of ions on their external parts [32]. As a result, the ions cannot penetrate the internal parts of the lesion, and the subsurface remains porous [30,31,33].

Recent studies have emphasized the effectiveness of combining various treatment techniques to manage WSLs, although without specific clinical guidelines. These integrated approaches combine remineralizing, micro-invasive, and preventive strategies to enhance both functional and aesthetic outcomes. The combined use of fluoride varnish, ozone, and octenidine mouthwash [34] significantly reduced the incidence of WSLs, particularly during orthodontic treatments, compared to the use of fluoride varnish alone. Infiltrating resin combined with chlorhexidine varnish showed a greater ability to slow the progression of advanced lesions (ICDAS 3), while also offering antimicrobial benefits [35]. Similarly, the combination of infiltrating resin and fluoride varnish was more effective in reducing caries progression than using varnish alone [34,36]. Remin Pro^TM^ and Remin Pro Forte^TM^ (VOCO GmbH, Cuxhaven, Germany), two remineralizing gels containing hydroxyapatite and fluoride, showed a significant reduction in WSLs, an increase in mineral content and an aesthetic improvement of lesions [37]. Treatments based on CPP-ACP also showed good results in remineralization [38] and aesthetic improvement of WSLs, although their combination with fluoride varnish did not provide additional benefits compared to CPP-ACP alone [34].

These findings highlight the potential of combined treatment strategies to address the multifaceted nature of WSLs by harnessing the synergistic effects of remineralizing agents, antimicrobial measures, and micro-invasive techniques. By integrating these approaches, clinicians can achieve a more comprehensive management of WSLs, improving clinical efficacy and patient satisfaction while adhering to the principles of minimally invasive dentistry.

This systematic review gathers and critically compares the available literature on the efficacy of combining multiple treatment techniques for WSLs compared to using these techniques alone. It will focus specifically on studies that investigate the combined use of approaches such as resin infiltration with fluoride varnish, remineralizing agents like CPP-ACP alongside fluoride-based products, and other integrated strategies in order to respond to the following hypothesis: Combined techniques lead to better clinical and aesthetic outcomes for WSLs than single-treatment methods.

Null Hypothesis (H0): There is no significant difference in clinical or aesthetic outcomes between combined and single-treatment techniques for WSLs.

## 2. Methods

### 2.1. Study Sources

A thorough and systematic literature search was performed using the electronic databases PubMed, Scopus, and Cochrane to identify relevant articles on the treatment options for WSLs. Additionally, the reference lists of potentially eligible studies were examined to ensure that any relevant articles not captured in the initial database searches were also included. This systematic review was conducted following the PRISMA (Preferred Reporting Items for Systematic Reviews and Meta-Analyses) guidelines [39] and the review protocol was registered in the OSF database (Registration DOI: https://doi.org/10.17605/OSF.IO/W74FM).

### 2.2. Search Strategy

A research question has been formulated following the PICO strategy, which is denoted as P (population), I (intervention), C (comparison), O (outcome): P (Patient/Problem): Patients with white spot lesions; I (Intervention): the combination of two or more treatment techniques compared to the use of one technique alone; C (Comparison): Comparison of different treatment techniques; O (Outcome): Aesthetic improvements or reduction in the visibility of the lesions; S (Study Type): Systematic review.

Below is the defined PICO question:

Which clinical approaches combined or isolated (C) influence the treatment and prevention effectiveness (O) of white spot lesions (I) in humans (P) in the last ten years (T)?

The search strategy was developed for the PubMed database and adapted for each database consulted. The results from the different databases were cross-checked to identify and eliminate duplicates. The search was conducted in July 2024, using the following keywords: (white spot lesions [Title/Abstract]) AND (treatment), and only articles from the past 10 years were selected. Studies were chosen based on the following inclusion criteria: studies conducted on humans, published in English or Portuguese between 2014 and 2024, and focused on a combination of several WSLs treatment techniques, with full text available. Systematic reviews of clinical trials, in vitro studies, critical/narrative reviews, and studies evaluating only one technique—not a combination of several techniques—for treating WSLs were excluded.

The selection of studies was conducted in three steps. First, two independent researchers (PL and ML) scanned relevant articles that fit the study criteria by analyzing the titles and abstracts. Disagreements between the two reviewers were resolved by discussion with a third author (RFP), who facilitated a consensus through mutual evaluation and deliberation. Cohen’s Kappa test was performed to assess the inter-rater reliability between the two reviewers, quantifying the level of agreement beyond chance. The Rayyan Intelligent Systematic Review Platform was utilized to support the systematic review process [39].

The second step involved evaluating the abstracts and non-excluded articles that met the eligibility criteria during the abstract review. The selected articles were read and analyzed individually in relation to the purpose of this study.

Finally, the qualified articles received a study nomenclature. The information collected during data extraction included the title of the article, year of publication, authors, study design, type of participants, number of participants, age of participants, type of treatment, duration of clinical follow-up, and evaluated outcomes.

The methodological quality of the included studies was assessed using the Revised Cochrane Risk of Bias Tool for Randomized Trials (RoB 2.0 [40]), including specific considerations for cluster-randomized trials, as outlined by the Cochrane Database of Systematic Reviews (2016).

This analysis is based on five domains: (1) Bias arising from the randomization process, (2) Bias due to deviations from intended interventions, (3) Bias due to missing outcome data, (4) Bias in measurement of the outcome, and (5) Bias in selection of the reported result [41]. Each domain was evaluated using one of the following judgments: low risk, some concerns, and high risk. The overall judgment for each article was determined by the most prevalent judgment across the domains.

## 3. Results

### 3.1. Characteristics of the Studies

**Figure 1:**
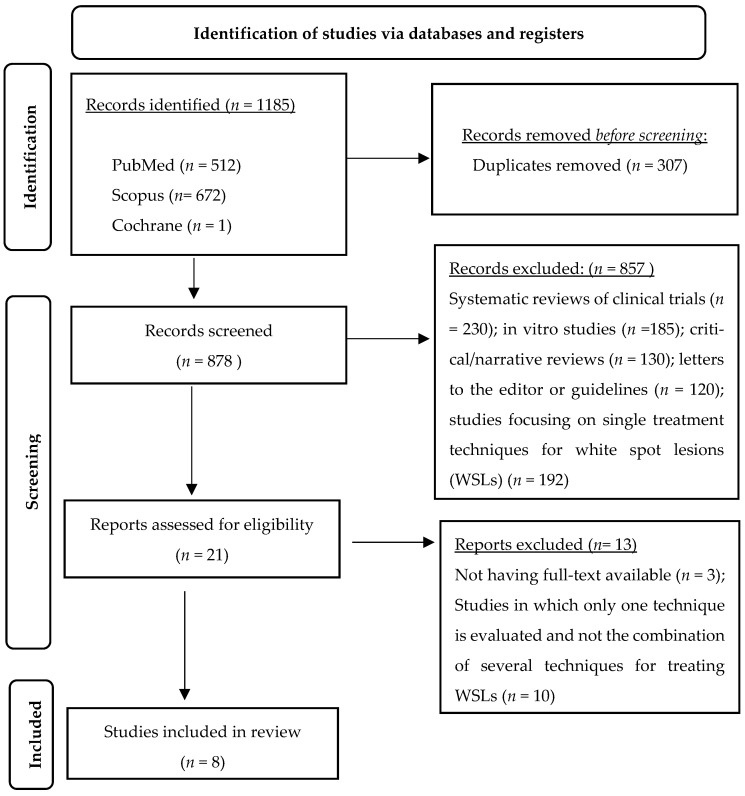
Overview of article selection procedure according to PRISMA guidelines.

The initial search yielded 1185 articles: 512 from PubMed, 672 from Scopus, and 1 from Cochrane. After 307 duplicates were removed, 878. articles were considered, retrieved, and assessed for screening. After an initial screening based on the title and abstract, 857 articles were excluded for the following reasons: (1) systematic reviews of clinical trials, (2) in vitro studies, (3) critical or narrative reviews, (4) letters to the editor or guidelines, and (5) studies that evaluated only one treatment technique for WSLs. A total of 21 articles were then selected for full reading. Of these, 13 studies were excluded because (1) the full text was not available or (2) they focused solely on one treatment technique without considering combinations of techniques. In the end, 8 studies were included in the review. The study selection process is described in Figure 1, and the relevant data gathered from the retrieved studies are represented in Table 1.

Table 1 summarizes the main characteristics of the eight studies included in this systematic review. All studies followed an experimental design, with the majority being randomized controlled trials (RCTs). One study (Table 1, entry 4) employed a split-mouth randomized design, which allows for intra-subject comparison, thereby enhancing control over individual variability in lesion development. The participants consisted of children, adolescents, and young adults aged 7 to 50 years. The included studies evaluated a range of treatment techniques: remineralizing gels (Remin Pro, Remin Pro Forte, MI Paste Plus; *n* = 6), resin infiltration (*n* = 2), Fluoride Varnish (*n* = 2), chlorhexidine varnish (*n* = 1), ozone therapy (*n* = 1). All eight studies involved combination approaches using two or more of these techniques. The duration of treatment was heterogeneous, ranging from a minimum of 10 days (Table 1, item 7) to a maximum of 12 months (Table 1, items 2, 3, and 5).

The main results can be summarized as follows:

Remineralizing gels: Formulations that combine bioactive ingredients such as fluoride, hydroxyapatite, and xylitol have shown a significant reduction in white spot lesions (WSLs), enhancing the mineral content and aesthetics of tooth surfaces (Table 1, items 1, 6, and 7). In particular, Remin Pro Forte, enriched with turmeric and ginger extracts, achieved significantly greater reductions in WSL area and mineral loss compared to conventional Remin Pro (Table 1, entry 1), suggesting that the additional bioactive ingredients may enhance remineralization efficacy. Infiltrating resin combined with fluoride varnish: Studies evaluating this combination have reported a more significant reduction in caries progression compared to the use of fluoride varnish alone (Table 1, items 4 and 5).

The combination of ozone, fluoride varnish, and octenidine mouthrinse resulted in a 75% lower incidence of new WSLs compared to the control group over 12 months (Table 1, item 3).

Studies using CPP-ACP-based creams, either alone or in combination with fluoride varnish, demonstrated significant improvements in the appearance and mineralization of white spot lesions (WSLs) (Table 1, items 6 and 8). However, fluoride varnish alone did not exhibit significant additional benefits compared to the control group in certain studies (Table 1, entry 8).

Infiltrating resin combined with other techniques has proven to be particularly effective in controlling advanced lesions (ICDAS 3) and halting caries progression (Table 1, item 4).

Remineralizing gels containing CPP-ACP or hydroxyapatite provide effective alternatives to traditional fluoride, showing strong remineralization capacity and aesthetic enhancements (Table 1, entries 6 and 7).

Overall, combination therapies achieved superior results compared to single-treatment approaches, as reflected by greater reductions in lesion size, enhanced mineral gain, and improved aesthetic appearance.

### 3.2. Risk of Bias Analysis

Table 2 presents the risk of bias analysis using the RoB tool [46]. This tool involves evaluating articles across five different domains, focusing on studies involving animals or humans, as well as other studies that require ethical approval. The first domain assesses bias arising from the randomization process. In this domain, seven articles exhibit a low risk of bias, suggesting that the randomization process was adequately reported and implemented. However, Heravi et al. (2018) [38] raised some concerns because, although the study claims that participants were randomly allocated, there is limited information regarding the method used for sequence generation and allocation concealment, which raises potential doubts about the true randomness of the process.

Domain two refers to bias due to deviations from the intended intervention. In this domain, seven articles demonstrate a low risk of bias, indicating that participants generally adhered to the assigned treatments with minimal deviations. However, Ebrahimi et al. (2017) [44] raised some concerns, as there is no explicit mention of whether adherence to the intervention was fully monitored or if deviations were accounted for in the analysis, leaving some uncertainty about the study’s control over protocol deviations.

Regarding the third domain, bias due to missing outcome data, five articles showed a low risk of bias, indicating that the studies either had complete follow-up or employed appropriate methods to address missing data. However, Ebrahimi et al. (2017) [44], Grocholewicz et al. (2022) [34], and Güçlü et al. (2016) [45] raised some concerns. Ebrahimi et al. (2017) [44] did not clarify whether all randomized participants were included in the final analysis. Grocholewicz et al. (2022) [34] had a 12-month follow-up, which increases the likelihood of patient dropout, yet no information was provided on how missing data were managed. Güçlü et al. (2016) [45] had a small sample size, and while dropouts are not explicitly mentioned, this raises concerns about potential data loss impacting the final results. These issues may affect result interpretation. Unaddressed missing data can limit generalizability and reduce confidence in outcomes.

Domain four evaluates bias in the measurement of outcomes. In this category, four articles have a low risk of bias, indicating that outcome assessment methods were clearly defined and consistently applied. However, Turska-Szybka et al. (2016) [43], Heravi et al. (2018) [38], Ebrahimi et al. (2017) [44], and Grocholewicz et al. (2022) [34] raised some concerns. Turska-Szybka et al. (2016) [43] did not clarify whether the outcome assessors were blinded, which may introduce detection bias. Heravi et al. (2018) [38] utilized subjective visual assessments, which increases the potential for measurement bias. Ebrahimi et al. (2017) [44] employed photographic analysis, which, despite being standardized, may introduce variability in interpretation. Grocholewicz et al. (2022) [34] assessed WSLs visually without specifying whether multiple examiners were involved to ensure consistency.

In the last domain, which evaluates bias in the selection of reported results, six articles demonstrate a low risk of bias, indicating that outcomes were pre-specified and all results were consistently reported. However, Turska-Szybka et al. (2016) [43] and Ebrahimi et al. (2017) [44] raised some concerns. Turska-Szybka et al. (2016) [43] lacked clarity on whether all measured outcomes were fully reported, which raises concerns about selective reporting. Ebrahimi et al. (2017) [44] did not explicitly state whether all planned analyses were conducted, leaving room for potential reporting bias.

Overall, this analysis indicates that seven of the eight articles included in the study present a low risk of bias, while Ebrahimi et al. (2017) [44] raised some concerns across multiple domains. These concerns primarily arose from uncertainties in protocol adherence, missing outcome data, potential measurement bias, and a lack of clarity regarding selective reporting. Although the remaining studies showed minor concerns in specific areas, they generally maintain a low overall risk of bias, reinforcing the reliability of their findings.

## 4. Discussion

WSLs are among the earliest signs of enamel demineralization and are particularly prevalent in patients undergoing orthodontic treatment or those with inadequate oral hygiene [11]. These lesions occur when the balance between remineralization and demineralization is disturbed, resulting in mineral loss within the enamel structure. Although WSLs do not always progress to cavitated carious lesions, their presence suggests an increased risk for further demineralization and caries development [42]. In addition to their clinical significance, WSLs can have a significant aesthetic impact, leading to patient dissatisfaction, especially in pediatric and adolescent populations [37]. Given their multifactorial etiology, managing WSLs requires a comprehensive approach that includes preventive strategies, remineralization therapies, and minimally invasive treatments.

Due to the high prevalence of WSLs and their clinical implications, this systematic review aimed to evaluate different WSLs treatments, either as single interventions or in combination. The results revealed notable differences between individual treatments and their synergistic combinations. Notably, combinations such as resin infiltration with fluoride varnish or ozone therapy with fluoride showed enhanced outcomes, suggesting that certain protocols may be more effective than monotherapies. Therefore, the findings rejected the hypothesis of this study.

### 4.1. Remineralizing Gels

Fluoride-based remineralizing gels, such as Remin Pro and Remin Pro Forte, demonstrate significant efficacy in managing WSLs. These formulations contain bioactive ingredients like hydroxyapatite, xylitol, and fluoride, which enhance enamel remineralization and improve aesthetic outcomes. A study by Mennatallah Atef Aboulnaga et al. (2022) [37] found that Remin Pro Forte, enriched with turmeric and ginger extracts, provided superior remineralization compared to conventional Remin Pro. This aligns with research suggesting that bioactive compounds like turmeric may aid in enamel repair due to their anti-inflammatory and antibacterial properties. These components not only facilitate enamel repair but may also reduce bacterial activity within lesions. The use of such enhanced remineralizing agents could offer additional benefits compared to fluoride alone, particularly in minimizing bacterial activity within WSLs [38,42,44,47,48,49].

### 4.2. Fluoride Gel Therapies

Fluoride remains a cornerstone in the treatment of WSLs, promoting remineralization and reducing demineralization. Peter Rechmann et al. (2017) [42] examined the use of fluoride toothpaste (1100 ppm), MI Paste Plus (CPP-ACP with fluoride), and MI Varnish. Their findings indicated no significant difference in enamel decalcification scores between the experimental and control groups at 12 months, suggesting that fluoride varnish alone may not be sufficient to prevent WSLs in orthodontic patients. Other studies support this, emphasizing that while fluoride is effective in strengthening enamel, its preventive efficacy is enhanced when combined with other remineralizing agents [30,31,33,36,42,45].

### 4.3. Ozone, Fluoride, and Octenidine

Grocholewicz et al. (2022) [34] analyzed the combination of fluoride varnish, ozone therapy, and octenidine mouthwash, demonstrating a significantly lower incidence of new WSLs compared to fluoride varnish alone. The antimicrobial action of ozone and octenidine likely disrupts biofilm and reduces acid-producing bacteria, creating a favorable environment for enamel remineralization. These findings support the synergistic use of antimicrobial agents and fluoride, especially in high-risk orthodontic patients. This aligns with findings that ozone therapy can effectively manage early carious lesions by inhibiting cariogenic bacteria (Urquhart et al., 2019 [32]). Therefore, the integration of ozone with fluoride-based therapies may represent a promising approach for high-risk orthodontic patients [34].

### 4.4. Resin Infiltration and Fluoride Varnish

Resin infiltration, especially when combined with fluoride varnish, has demonstrated better outcomes than fluoride varnish alone. Turska-Szybka et al. (2016) [43] showed that this combination significantly reduced the progression of early carious lesions. Rai et al. (2016) [35] further explored resin infiltration with and without chlorhexidine varnish, revealing that adding chlorhexidine enhanced caries control in more advanced lesions (ICDAS 3). Resin infiltration is recognized for its capacity to aesthetically mask WSLs while preventing lesion progression, making it a preferred option for both functional and cosmetic results [16,25,43,50,51].

Studies assessing MI Paste Plus (CPP-ACP with fluoride) and other CPP-ACP formulations found significant improvements in WSL remineralization. Heravi et al. (2018) [38] and Ebrahimi et al. (2017) [44] reported considerable reductions in WSLs areas, along with increased mineral content and enhanced aesthetics. However, their findings indicated that the combination of CPP-ACP and fluoride did not necessarily produce better outcomes compared to CPP-ACP alone. This aligns with research suggesting that CPP-ACP is highly effective in stabilizing calcium and phosphate ions at the enamel surface, promoting remineralization independent of fluoride [27,49]. It is important to clarify that CPP-ACP can promote remineralization independently of fluoride but may also act synergistically with fluoride for enhanced results.

For more advanced lesions (ICDAS 3), resin infiltration has shown greater efficacy in controlling lesion progression compared to fluoride varnish alone. Rai et al. (2016) [35] highlighted the advantages of combining resin infiltration with chlorhexidine varnish, underlining the significance of antimicrobial agents in managing deeper lesions. The minimally invasive nature of resin infiltration positions it as a valuable alternative to restorative interventions, preserving enamel structure while halting lesion development [21,35,36,52].

The findings of this systematic review emphasize the significance of combination therapies in managing WSLs. While fluoride remains a crucial element, its effectiveness is enhanced when combined with remineralizing gels, antimicrobial treatments, or resin infiltration. However, the success of these interventions is influenced by factors such as lesion severity, patient compliance, and follow-up duration. Differences in study protocols, including treatment durations and assessment methods, complicate direct comparisons between trials. Furthermore, some studies had relatively brief follow-up periods, which limit the ability to evaluate long-term outcomes [1,3,6,7]. The present study is enlightening on the combined approach of the described therapies. Supporting that combining resin infiltration with fluoride varnish gel is a consistent combined treatment for WSLs [43]. On the other hand, fluoride gel therapies combined with ozone therapy, and octenidine mouthwash therapies are more effective in WSLs treatment than fluoride gel alone [34].

Future research should prioritize optimizing treatment protocols, identifying the most effective combinations for various patient profiles, and assessing the long-term stability of treatment outcomes. Studies with larger sample sizes and standardized methodologies are essential to establish evidence-based guidelines for WSLs management. Additionally, integrating novel biomaterials, such as peptide-based remineralizing agents, may provide further benefits in enhancing enamel repair.

## 5. Conclusions

While prevention of WSLs remains the cornerstone method—through effective oral hygiene, fluoride gel use, and dietary counseling—advances in minimally invasive therapies with gels have opened new doors for aesthetic and functional recovery. The combination of remineralizing agents like CPP-ACP and fluoride, along with techniques such as resin infiltration, has shown promising synergistic effects, offering clinicians a more comprehensive approach. Ultimately, tailoring the intervention to each patient’s needs and lesion severity ensures more predictable and satisfactory outcomes.

## Figures and Tables

**Table 1:** Relevant data gathered from the retrieved studies.

Author/ Year	Study Design	Participants (nr/age)	Intervention Time	Treatment Techniques in Study	Results	Conclusions
Mennatallah Atef Aboulnaga et al., 2022 [37]	RCT	40 participants, aged 15–25	3 months	Remin Pro (SF—1450 ppm, hydroxyapatite, xylitol) and Remin Pro Forte (SF—1450 ppm, hydroxyapatite, xylitol, ginger extracts, and curcuma)	Both were effective, with Remind Pro Forte showing superior performance.	The combination of additional bioactive ingredients, including ginger extracts and curcuma, in Remin Pro Forte provides enhanced remineralization, making it more effective for managing WSLs.
Peter Rechmann et al., 2017 [42]	RCT	40 participants, age range 11–26 years	12 months	Experimental group: twice-daily fluoride toothpaste, daily MI Paste Plus MIPP), quarterly MI Varnish (MIV) application	No significant differences in enamel decalcification or caries detection (ICDAS) scores between experimental and control groups at 12 months.	Varnish did not significantly reduce the incidence of WSLs during fixed orthodontic treatment. However, they delivered fluoride effectively.
Grocholewicz et al., 2022 [34]	RCT	150 patients, aged 16–50 years	12 months	Fluoride varnish, ozone therapy, octenidine mouthrinse	Lower incidence of WSLs with combined treatment techniques.	Combination of fluoride varnish, ozone, and octenidine reduced WSLs compared to control group.
Rai et al., 2016 [35]	Split-mouth randomized study	45 children, age range 7–15 years	9 months	Resin infiltration with or without chlorhexidine varnish	Slower progression of caries in test group (resin infiltration + chlorhexidine varnish).	Resin infiltration with chlorhexidine varnish over resin infiltration alone, improving caries control in more advanced lesions (ICDAS 3).
Turska-Szybka et al., 2016 [43]	RCT	81 children, 18–71 months	1 year	Resin infiltration with fluoride varnish vs. fluoride varnish only	Significantly reduced progression of early caries lesions with resin infiltration.	Resin infiltration combined with fluoride varnish is more effective than fluoride varnish alone in arresting caries.
Heravi et al., 2018 [38]	RCT	36 participants (13–23 years)	12 weeks	MI Paste Plus (containing CPP-ACPF), Remin Pro (containing hydroxyapatite and fluoride), control	Significant reduction in WSL area, increased mineral content, and improved aesthetics for MI Paste Plus and Remin Pro groups.	The combination of CPP-ACP and fluoride in MI Paste Plus, and hydroxyapatite, fluoride, and xylitol in Remin Pro provide effective remineralization and improved aesthetics for WSLs.
Ebrahimi et al., 2017 [44]	RCT	80 children (7–12 years)	10 days	MI Paste Plus (containing CPP-ACPF), Remin Pro (containing hydroxyapatite and fluoride), 2% SF gel	All remineralizing agents significantly reduced the area and increased the mineral content of WSLs. The control group showed no significant improvement.	The combination of bioactive ingredients in MI Paste Plus and Remin Pro was as effective as 2% SF, making them suitable alternatives for managing WSLs in children.
Güçlü et al., 2016 [45]	RCT	21 children (8–15 years)	12 weeks	Application of 10% CPP-ACP paste and 5% SF varnish	CPP-ACP and its combination with fluoride varnish significantly improved visual appearance and remineralization of WSLs. Fluoride varnish alone showed no additional advantage.	Twice-daily application of CPP-ACP significantly improves WSLs’ appearance and mineralization, making it effective as a standalone treatment.

RCT—randomized clinical trial; WSLs—white spot lesions; SF—sodium fluoride; CPP-ACP—Casein phosphopeptide–amorphous calcium phosphate nanocomplex.

**Table 2:** Risk of bias analysis according to the RoB tool (Cochrane Library). D1: Bias arising from the randomized process, D2: Bias due to deviations from intended intervention, D3: Bias due to missing outcome data, D4: Bias in measurement of the outcome, and D5: Bias in selection of the reported result. Judgment: high (red), some concerns (yellow), and low (green).

Study (Author, Year)	D1:	D2:	D3:	D4:	D5:	Overall Risk of Bias
Aboulnaga et al., 2022 [37]	🟢 Low	🟢 Low	🟢 Low	🟢 Low	🟢 Low	🟢 Low
Rechmann et al., 2017 [42]	🟢 Low	🟢 Low	🟡 Some Concerns	🟢 Low	🟡 Some Concerns	🟢 Low
Grocholewicz et al., 2022 [34]	🟢 Low	🟢 Low	🟡 Some Concerns	🟡 Some Concerns	🟢 Low	🟢 Low
Rai et al., 2016 [35]	🟢 Low	🟢 Low	🟢 Low	🟢 Low	🟢 Low	🟢 Low
Turska-Szybka et al., 2016 [43]	🟢 Low	🟢 Low	🟢 Low	🟡 Some Concerns	🟡 Some Concerns	🟢 Low
Heravi et al., 2018 [38]	🟡 Some Concerns	🟢 Low	🟢 Low	🟡 Some Concerns	🟢 Low	🟢 Low
Ebrahimi et al., 2017 [44]	🟢 Low	🟡 Some Concerns	🟡 Some Concerns	🟡 Some Concerns	🟢 Low	🟡 Some Concerns
Güçlü et al., 2016 [45]	🟢 Low	🟢 Low	🟡 Some Concerns	🟢 Low	🟢 Low	🟢 Low

## Data Availability

Dataset available on request from the authors.

## Abbreviations

The following abbreviations are used in this manuscript:

WSLs	White Spot Lesions
CPP-ACP	Casein Phosphopeptide–Amorphous Calcium Phosphate
ICDAS	International Caries Detection and Assessment System
ECM	Electronic Caries Monitor
QLF	Quantitative Light-Induced Fluorescence
TEGDMA	Triethylene Glycol Dimethacrylate
DEJ	Dentin–Enamel Junction
MI Paste Plus	Casein Phosphopeptide–Amorphous Calcium Phosphate with Fluoride
PRISMA	Preferred Reporting Items for Systematic Reviews and Meta-Analyses
RCT	Randomized Clinical Trial
